# Getting to the Roots: A Developmental Genetic View of Root Anatomy and Function From Arabidopsis to Lycophytes

**DOI:** 10.3389/fpls.2018.01410

**Published:** 2018-09-25

**Authors:** Frauke Augstein, Annelie Carlsbecker

**Affiliations:** ^1^Department of Organismal Biology, Physiological Botany and Linnean Centre for Plant Biology in Uppsala, Uppsala University, Uppsala, Sweden

**Keywords:** roots, plant evo-devo, plant development, plant anatomy and morphology, patterning, gene regulatory network

## Abstract

Roots attach plants to the ground and ensure efficient and selective uptake of water and nutrients. These functions are facilitated by the morphological and anatomical structures of the root, formed by the activity of the root apical meristem (RAM) and consecutive patterning and differentiation of specific tissues with distinct functions. Despite the importance of this plant organ, its evolutionary history is not clear, but fossils suggest that roots evolved at least twice, in the lycophyte (clubmosses and their allies) and in the euphyllophyte (ferns and seed plants) lineages. Both lycophyte and euphyllophyte roots grow indeterminately by the action of an apical meristem, which is protected by a root cap. They produce root hairs, and in most species the vascular stele is guarded by a specialized endodermal cell layer. Hence, most of these traits must have evolved independently in these lineages. This raises the question if the development of these apparently analogous tissues is regulated by distinct or homologous genes, independently recruited from a common ancestor of lycophytes and euphyllophytes. Currently, there are few studies of the genetic and molecular regulation of lycophyte and fern roots. Therefore, in this review, we focus on key regulatory networks that operate in root development in the model angiosperm Arabidopsis. We describe current knowledge of the mechanisms governing RAM maintenance as well as patterning and differentiation of tissues, such as the endodermis and the vasculature, and compare with other species. We discuss the importance of comparative analyses of anatomy and morphology of extant and extinct species, along with analyses of gene regulatory networks and, ultimately, gene function in plants holding key phylogenetic positions to test hypotheses of root evolution.

## Fossils, Phylogenies, and Developmental Genetics in Tracing the Evolution of Roots

Roots anchor plants to the ground, and their growth patterns allow exploration of the soil while their specific morphology and anatomy are adapted for efficient uptake of water and mineral nutrients. The evolution of deeply penetrating roots dramatically altered living conditions on Earth. Their activity is capable of weathering rocks resulting in accessible silicate material which reacts with and binds carbon dioxide thereby reducing it from the atmosphere (Raven and Edwards, 2001; Pires and Dolan, 2012). Roots are essential for the formation of complex soils and allow intimate symbiotic relationships with fungi and bacteria (Raven and Edwards, 2001). Thus, both the abiotic and biotic environment were altered with the evolution of roots and plant roots remain essential for our ecosystems. Furthermore, optimal plant root behavior in response to external conditions such as mineral nutrient and water availability is essential for crop survival and yield (Ahmed et al., 2018). Despite their importance, the evolutionary history of roots is currently not clearly understood and the developmental genetic regulation of root traits is known in considerable detail essentially only in the model plant *Arabidopsis thaliana* (Arabidopsis), although knowledge from other angiosperm (primarily crop) species is rapidly catching up.

All extant vascular plants have true roots (with few exceptions) distinguished by positive gravitropism and a root cap protecting a meristem that allows continuous growth. Roots have root hairs extending the surface area for efficient water and mineral uptake, and a ground tissue that often harbors an inner specialized endodermal cell layer controlling uptake into the vascular stele. The stele generally has a primitive protostele organization with central xylem (Raven and Edwards, 2001). Although being united by these characteristics the fossil record along with certain developmental features (see below) strongly suggest that roots evolved independently several times, implying that several root specific structures convergently evolved multiple times. This raises the question if distinct genetic components were employed for similar functions or if these multiple independent events involved adoption of related genetic circuits present in the ancestor of these plants. If so, they would display deep homology, i.e., when the structures themselves are analogous but regulated by homologous genes (Scotland, 2010). Exploring the gene regulatory networks underlying root development in phylogenetically informative species both at great evolutionary distances, and in closely related species with similar or distinct anatomies, will give valuable information on the genetic tool kit(s) employed in root formation, and, potentially, in their evolution. Here, we review current hypotheses of how roots might have evolved, we describe selected key genetic circuits essential for different aspects of root development, with a focus on meristem maintenance and anatomy, and discuss how such information can help testing hypotheses for root evolution.

## The Origin and Anatomical Diversity of Roots

**FIGURE 1:**
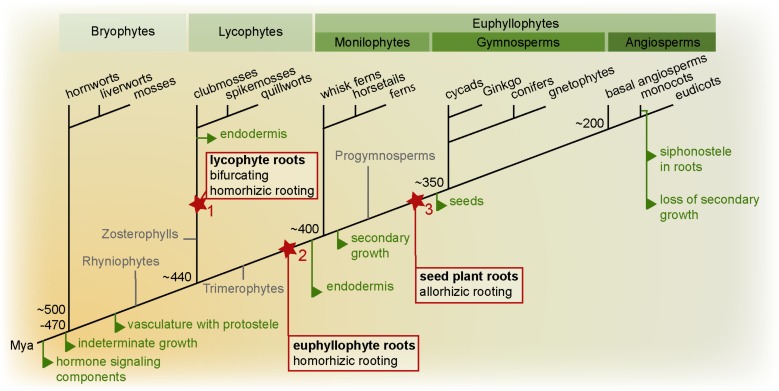
Land plant phylogeny highlighting key root evolutionary events. Important evolutionary innovations are mapped on a phylogenetic tree of land plants (simplified tree after Puttick et al., 2018). True roots are hypothesized to have evolved at least twice, in the lycophyte (1) and euphyllophyte lineage (2). In the lycophyte lineage, the root meristem is bifurcating, while in the euphyllophyte lineage, lateral roots emerge from the endodermis or pericycle. In lycophytes and monilophytes, the root develops as an adventitious organ “homorhizoic roots,” while in seed plants, the root and shoot meristems are bipolar, and the root is said to be “allorhizoic” (although seed plants also form adventitious roots). It is currently unclear how homorhizoic and allorhizoic roots relate to each and whether the evolution of a primary root in the seed plant lineage can be seen as a third root-evolution event (3) (see Liu and Xu, 2018). Evolution of homorhizoic and allorhizoic roots are marked in red, other major root evolutionary events are indicated in green. Mya: million years ago.

Rooting structures are found in all land plants; in the form of rhizoids (uni- or multicellular filamentous rooting structures emanating from non-root organs) in the free-living gametophytes of bryophytes, lycophytes, and monilophytes, and as true roots with elaborate tissues, as described above or with some variation, in the sporophytes of extant vascular plants (Raven and Edwards, 2001). This seemingly indicates a monophyletic origin of true roots. However, the fossils found of early vascular plants and certain aspects of how roots develop in different lineages, instead suggest a considerably more complex evolutionary history of roots (**Figure 1**). At the time when the first root-like structures appear in the fossil record, sporophytic plants had indeterminately growing upright or crawling, rhizome-like, shoot axes, some with microphyllous leaves (Taylor et al., 2009; Kenrick and Strullu-Derrien, 2014). The Rhyniophytes found in the Rhynie chert from Early Devonian are among the earliest fossils found of sporophytes with rooting structures (Taylor et al., 2009). These distinct fossils had a system of shoot-like axes, either a pro-vasculature or a distinguishable xylem, but no roots as specified by a root meristem covered by a cap. Instead, they had rhizoids (otherwise only known from gametophytes) directly developed from the lower surfaces of the axes at places where they were growing horizontally, on top or just beneath the soil surface (Taylor et al., 2009; Kenrick and Strullu-Derrien, 2014; Hetherington and Dolan, 2018a). Thus, it is possible that these axes had adapted a specific genetic program normally responsible for gametophyte rhizoid formation, to allow something of a rooting function to these axes. Such a combination of rhizoids formed on a sporophytic axis is an early evolutionary elaboration not found in any extant plant (Hetherington and Dolan, 2018a), but it emphasizes the similarities of rhizoids and root hairs (single cell tubular epidermal outgrowths of true roots). Indeed, comparisons of the genetic regulation of bryophyte gametophyte rhizoid and angiosperm root hair formation have identified a number of homologous factors regulating the formation of the analogous tip-growing cells with rooting function (Menand et al., 2007; Tam et al., 2015; Honkanen et al., 2017, reviewed in Jones and Dolan, 2012; Honkanen and Dolan, 2016), suggesting that a pre-existing genetic network for rhizoid formation was co-opted in the sporophyte generation.

Thus, Rhyniophytes, stem group taxa for all extant vascular plants, lacked true roots. Interestingly, also fossils of stem groups of both lycophytes (Zosterophylls) and fern and seed plants (Trimerophytes) lacked true roots (Raven and Edwards, 2001; Taylor et al., 2009; Kenrick and Strullu-Derrien, 2014). Therefore, true roots likely evolved independently in these two major vascular plant branches. The earliest fossils with true roots have affinity to the lycophytes. Most of these had apparently indeterminate growth, a root cap-like structure, no cuticula, and branched dichotomously by bifurcation of the meristem and most likely lacked an endodermis. Intriguingly, a very early fossil of a lyopsid root meristem found in the Rhynie chert was recently described in great detail (Hetherington and Dolan, 2018b). While these roots had positive gravitropism and a promeristem that had set off cells for vascular, ground and epidermal tissues, they had no signs of having had a root cap. Hence, Hetherington and Dolan take this as evidence for step-wise acquisitions of key root traits during root evolution in the lycophyte linage. Indirectly, this further supports that such root traits must have convergently evolved independently in the other major vascular plant lineage, the euphyllophytes (Hetherington and Dolan, 2018b).

In other fossils of Early Devonian lycophytes, non-gravitropic thin root-like structures in position of leaves originated from shoot-like positively gravitropic axes (Matsunaga and Tomescu, 2016). Thus, these lycophyte roots formed as a novel type of organ, and this fossil further suggests that certain lycophyte roots may have co-opted positive gravitropism at a later evolutionary step. In other early fossils, roots developed from many different sites of the plant: from stems, leaves, or other places (Taylor et al., 2009; Hetherington and Dolan, 2017). Hence, the diversity in where roots appeared may even suggest that roots evolved multiple times among the then very diverse lycophytes. Still today, lycophyte (*Lycopodiales*, clubmosses; *Selaginelales*, spikemosses; *Isoetales*, quillworts) roots display ancestral characters such as meristem branching by bifurcation, and *Lycopodium* has no root endodermis, although other lycophytes develop an endodermis (Hetherington and Dolan, 2017). Indeed, even among extant lycophytes there is a large variation in root meristem morphology – some have elaborate meristems with multiple stem cells as in seed plants (see below), while others have only one apical cell dividing to give rise to all root tissues as in ferns – supporting the paleobotanical indications of multiple evolution of roots in the lycophyte lineage (Fujinami et al., 2017).

In contrast to the lycophytes, euphyllophytes do not branch by bifurcation, but from internal tissues (from the endodermis in ferns and pericycle in seed plants) proximally to the apical meristem. Similar to lycophytes, fern roots develop as adventitious outgrowths in relation to the longitudinal axis of the embryo, and form so called “homorhizoic” roots (Raven and Edwards, 2001). In fossils (e.g., *Archaeopteris*) of progymnosperms, from which the seed plants evolved, plants have been found where the root formed at the opposing end of the shoot apical meristem (SAM), referred to as “bipolar” or “allorhizoic” roots (Taylor et al., 2009). All seed plants are distinguished by having allorhizoic roots although it is not uncommon to also find roots developing from non-root organs such as stems or leaves. It is currently an unresolved issue if the allorhizoic seed plant root is homologous with the homorhizoic fern root.

Thus, the evolutionary history of roots is still not clear, but there is good evidence that roots appeared as multiple independent innovations in the two major lineages leading to extant lycophytes and euphyllophytes. One can envision different trajectories by which roots may have evolved: They may have appeared as an entirely new type of organ, perhaps as a modification of a lateral shoot organ, or they could have evolved as modifications of shoot-like axes, already harboring an apical meristem.

## The Root Apical Meristem and Auxin – Similarities and Differences in Angiosperms, Ferns, and Lycophytes

**FIGURE 2:**
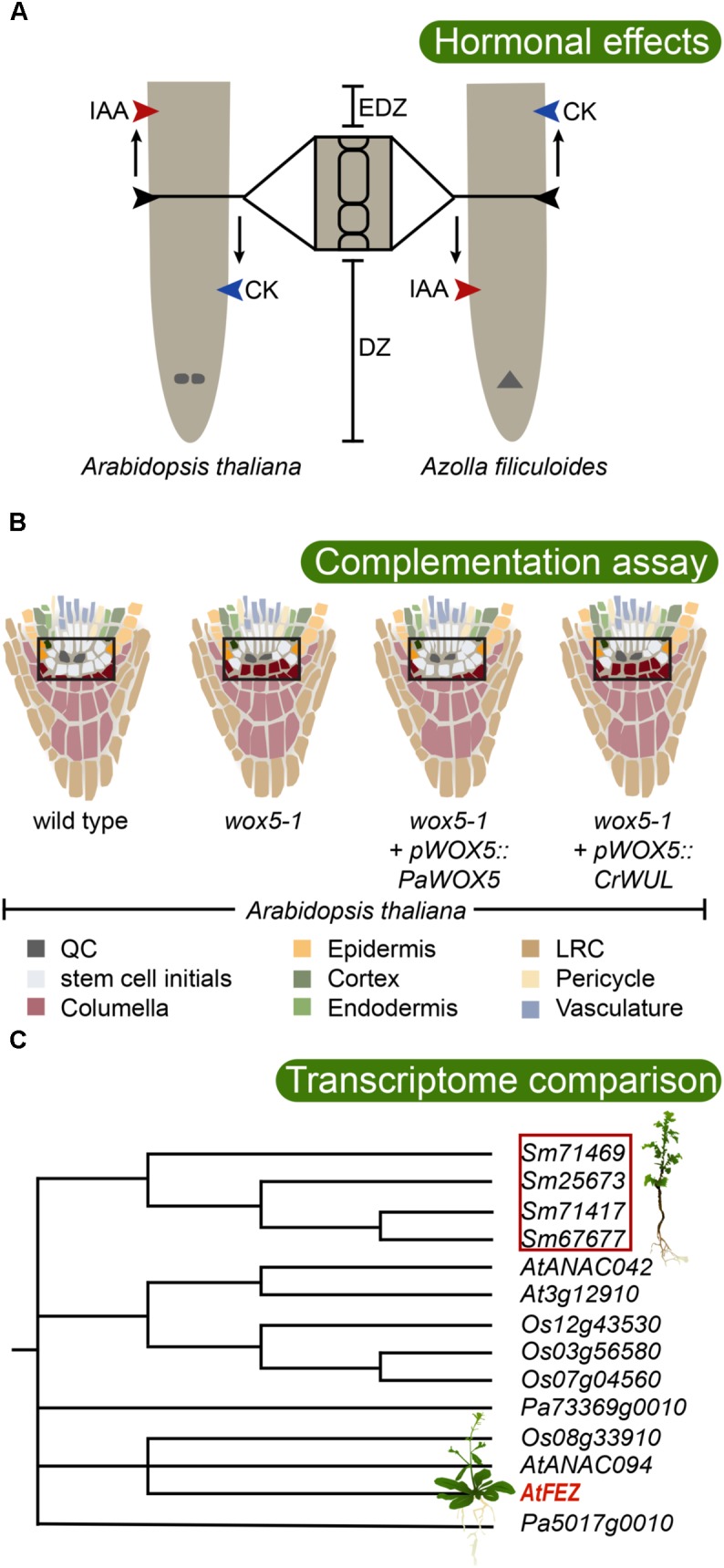
Testing the evolutionary conservation of root developmental regulators. **(A)** Root meristem development of both *Arabidopsis thaliana* (allorhizoic root) and *Azolla filiculoides* (homorhizoic root) is affected by the phytohormones auxin (IAA) and cytokinin (CK), but with opposite effects. While application of IAA increases root meristem size in Arabidopsis, it has a restricting effect in *A. filiculoides*. Contrary, CK inhibits root meristem growth in Arabidopsis, while it promotes it in *A. filiculoides* (de Vries et al., 2016). DZ, division zone; EDZ, elongation and differentiation zone. The QC is indicated in the Arabidopsis root, and the apical cell is indicated in the *A. filiculoides* root. **(B)** WOX5 is critical for maintaining the undifferentiated state of root apical meristem stem cells (Sarkar et al., 2007). Consistently, in the Arabidopsis *wox5-1* mutant, premature differentiation of columella stem cells is observed by accumulation of statoliths (indicated in purple). Introduction of the conifer *Picea abies WOX5* homolog *PaWOX5*, driven by the *WOX5* promoter, is able to restore the *wox5-1* phenotype, while the fern *Ceratopteris richardii WUS*/*WOX5* homolog *CrWUL*, driven by the *WOX5* promoter, cannot compensate for loss of WOX5 function (Zhang et al., 2017). This suggests that certain WOX5-specific properties evolved in the seed plant lineage. The stem cell niche is boxed, and colors inside of box are enhanced. **(C)** Transcriptome analysis revealed that a large number of angiosperm root meristem specific genes have homologs expressing in the root tip of the lycophyte *Selaginella moellendorffii* (Huang and Schiefelbein, 2015). In Arabidopsis, FEZ is required for LRC formation. The lycophyte root cap is an apparently analogous structure. Interestingly, *S. moellendorffii* expresses a set of FEZ-related genes in its roots suggesting the possibility of deep homology of factors regulating root cap development. The phylogenetic tree shows the well supported FEZ-clade, after Huang and Schiefelbein, 2015, including sequences from *Selaginella moellendorffii* (*Sm*), *Picea abies* (*Pa*), *Oryza sativa* (*Os*), and *Arabidopsis thaliana* (*At*). Poorly supported branchings are collapsed.

As discussed above, the evolution of roots was predated by shoots growing indeterminately. Thus, genetic circuits ensuring indeterminate growth must have been present, and may have been co-opted to allow indeterminate growth of roots. This could have occurred either by converting a shoot or by activating such a genetic circuit ensuring indeterminate growth elsewhere thereby triggering continuous growth *de novo*. Indeterminate growth is made possible by the activity of apical meristems that harbors pluripotent, constantly dividing cells. The activity of the meristem is ensured by stem cells (also called initial cells) that divide asymmetrically to give rise to cells that either undergo further divisions or begin to differentiate (Scheres, 2007). Stem cells for the specific tissue types of the root are found close to the root tip: distally (toward the tip) for the columella, proximally (shootward) for the stele and cortex/endodermis, and laterally distally for epidermis/lateral root cap (LRC). The proximal cells continue dividing within a division zone (DZ), until they reach a point in which division ceases and elongation begins. They then enter the elongation zone, and later the differentiation zone (collectively EDZ), where tissues are fully differentiated (**Figure 2A**; Dello Ioio et al., 2008; Perilli et al., 2012). The stem cells in the root apical meristem (RAM) are organized around mitotically inactive cells called quiescent center (QC) cells. The size of the QC (and the stem cell niche, i.e., QC + stem cells) varies substantially between species. Arabidopsis has a small meristem with only four QC cells. Within the monocots rice has 4–6, barley 30, and maize 500–1000 (Jiang et al., 2010; Kirschner et al., 2017). In a set of seminal cell laser ablation experiments, it was shown that if a QC cell in Arabidopsis is damaged, the neighboring columella stem cell begins to accumulate gravity sensing amyloplasts, showing that it is undergoing differentiation and suggesting that the QC sends a signal to surrounding stem cells to keep them undifferentiated (van den Berg et al., 1997).

Most of our current knowledge of the developmental genetic regulation of RAM establishment and maintenance comes from studies in Arabidopsis. Polar transport of auxin from the shoot down the root, *via* PIN auxin efflux carriers, creates an auxin maximum at the root tip which is needed for the maintenance of the stem cell niche. Here, auxin transport creates a “fountain” where auxin is refluxed up along the epidermis determining the location of the transition from DZ to EDZ (Blilou et al., 2005). In this process, auxin transport generates a concentration gradient over the meristem (Grieneisen et al., 2007). This auxin gradient determines the distribution of APETALA2-like PLETHORA (PLT) TFs that dose-dependently govern the extent of cell proliferation over the meristem (Galinha et al., 2007; Mähönen et al., 2014; Santuari et al., 2016). Considering the possibility that the RAM may have evolved from a pre-existing SAM it is interesting to note that the focused auxin maximum in the stem cells of the RAM is conceptually different from the SAM, where auxin maxima instead converge at the SAM periphery to promote lateral organ (e.g., leaf) formation (reviewed by Su et al., 2011). Auxin is transported away from these maxima and positive feedback from auxin concentration on auxin transport capacity canalizes auxin to narrow strands, which triggers procambium formation and xylem differentiation, while being transported toward the root. Within the SAM, cytokinin maintains cell division. In the root, however, cytokinin instead promotes cells to enter the EDZ and begin to differentiate. Therefore, a premature EDZ formation and smaller root meristem are the results of cytokinin application to roots (**Figure 2A**; Dello Ioio et al., 2008). Monocots appear to respond in a similar manner to applied cytokinin as the root meristem of barley (*Hordeum vulgare*) becomes considerably smaller after application of cytokinin (Kirschner et al., 2018), suggesting conservation in hormonal regulation of root meristem size among angiosperms. Indeed, auxin and cytokinin-mediated regulation of plant development, including regulation of indeterminate growth, is strongly conserved and important also in bryophytes (Coudert et al., 2015; Flores-Sandoval et al., 2015; Bowman et al., 2017; Mutte et al., 2018; Thelander et al., 2018). Hence, this predates the evolution of vascular plants, and the evolution of roots, and we should therefore expect auxin and cytokinin to play important roles in lycophyte and fern root development.

In the water fern *Azolla filiculoides* which has a DZ and EDZ similar to an Arabidopsis root, application of cytokinin promotes cell division and enlarges the meristem, while auxin reduces it (**Figure 2A**; de Vries et al., 2016). Thus, the response in the *A. filiculoides* root to auxin and/or cytokinin is distinctly different from the response in Arabidopsis, and the fern root response resembles Arabidopsis SAM rather than RAM, with cytokinin promoting and auxin restricting meristem growth. These findings may support the hypothesis that euphyllophyte roots originated as postembryonically branching shoot structures, and that the seed plant primary root therefore is conceptually different and potentially non-homologous with the fern root (de Vries et al., 2016). Currently, it is not clear if the fern RAM requires an auxin maximum for its establishment and maintenance, but the inability of auxin to trigger lateral root formation in *Ceratopteris richardii*, which normally produces lateral roots from an internal endodermal cell layer (Hou et al., 2004), suggests that auxin plays different roles in fern roots compared to seed plant roots.

On the other hand, in the lycophyte *Selaginella kraussiana*, application of auxin promoted, while cytokinin inhibited, dichotomous branching of the roots, although roots forming after hormonal treatment were morphologically distorted (Sanders and Langdale, 2013). Also, many genes active in the *Selaginella moellendorffii* root are related to auxin, reinforcing the importance for this hormone in lycophyte root development (Huang and Schiefelbein, 2015). Intriguingly, analysis of fossils of an arborescent isoetalean lycophyte suggests that while the young plant had a shoot meristem and a “foot,” after the meristem had bifurcated, one of the two meristems bent downward forming a “rhizomorph” – a shoot with a rooting function – thereby generating a plant with an apparently bipolar organization (Sanders et al., 2010). Analysis of the patterns of xylem strands in the isoetalean fossil suggests that auxin was transported from the shoot down to the “root”-part (Sanders et al., 2010). Hence, reversion of polar auxin transport towards a positively gravitropic meristem could indicate a mechanism by which a shoot meristem may have been converted to a root meristem. This interpretation is supported by extant *S. kraussiana* clearly exhibiting basipetal auxin transport in shoots (Sanders and Langdale, 2013). Thus, there is support both for evolution of roots as converted shoots, and as entirely novel organs. One may therefore imagine that this occurred by several different type of mechanisms, enforcing the need to genetically and functionally assess root development and auxin response, in multiple lycophyte and fern species.

## The Root Meristem: A Mirror Image of the Shoot Meristem?

Auxin and cytokinin as well as specific TFs are required for establishing and maintaining the RAM. Key TFs for maintenance of the QC and the stem cell niche are the PLTs (see above; Aida et al., 2004), the GRAS-type TFs SCARECROW (SCR) and SHORTROOT (SHR) (Sabatini et al., 2003), and the WUSCHEL-related homeobox5 (WOX5) (reviewed in Heyman et al., 2014). Interestingly, a recent study now link these factors, as both PLT and SCR were shown to interact with a third type of TF of the TCP type (Shimotohno et al., 2018). This PLT–SCR–TCP complex activates *WOX5* expression, which is required for keeping stem cells, in particular columella stem cells undifferentiated. While PLT and SCR have broader activity domains, the *WOX5* gene is specifically expressed in the QC (Sarkar et al., 2007). However, WOX5 acts non-cell autonomously because in the *wox5* mutant columella stem cells show signs of differentiation (**Figure 2B**; Sarkar et al., 2007). Hence, WOX5 may constitute the signal that was suggested by QC ablation experiments to emanate from the QC to maintain columella initial cells undifferentiated (van den Berg et al., 1997). The WOX5 protein has been shown to repress differentiation by forming a complex with a conserved repressor protein (Pi et al., 2015). *WOX5* expression is restricted to the QC by peptide signaling from differentiated cells in the columella. This involves the CLE40 peptide which signals through the Arabidopsis CRINKLY4 (ACR4) receptor kinase (Stahl et al., 2009). This regulation is interesting from an evolutionary point of view because the WOX5 paralog WUSCHEL (WUS), which is active specifically in the organizing center of the SAM and required to maintain its stem cells, is similarly regulated by paralogous peptides and receptors (Stahl and Simon, 2010). Here, the CLV3 peptide signals from the outer L1 layer to restrict WUS to the central SAM a few cell layers below. Thus, if the RAM and the SAM are maintained by homologous factors, is this suggesting a common evolutionary origin for the two meristems?

While little is known of the evolutionary history of PLT and SCR type TFs, the WOX5/WUS genes have been studied more intensively. Despite being active in the two different meristems, the protein function of WOX5 and WUS has been conserved, as shown by their interchangeability: WUS can restore a functional QC in a *wox5* mutant when directed by the *WOX5* promoter-sequence, and WOX5 can compensate for loss of WUS in a similar type experiment (Sarkar et al., 2007). Tracing the phylogeny of WUS/WOX5 genes revealed orthologs of both *WUS* and *WOX5* in both conifers and *Ginkgo*, but ferns have only one basal ortholog to these paralogous genes (Hedman et al., 2013). Thus, a gene duplication that gave rise to its co-orthologs *WUS* and *WOX5* likely took place in the lineage leading to the seed plants. In *C. richardii*, the single ortholog *CrWUL* marks pluripotent cells in the shoot apex and also the proximal part of the root meristem, albeit not the root apical cell nor the distal side where the root cap is located (Nardmann and Werr, 2012). Assessing the ability of the fern and gymnosperm homologs to rescue the Arabidopsis *wus* or *wox5* mutant phenotypes, Zhang et al. (2017) found that both gymnosperm *WUS* and *WOX5* homologs have conserved protein function, whereas the CrWUL protein was not able to replace neither WUS nor WOX5 (**Figure 2B**), unless it was specifically expressed in the columella initials. This shows that CrWUL has lost its cell-to-cell mobility, at least when expressed in Arabidopsis, but that it does have the possibility to interact in a similar molecular context and repress differentiation. In *C. richardii*, this is likely not relating to root cap development, as its activity domain is proximal to the apical cell of the meristem (Nardmann and Werr, 2012). Hence, this suggests that the central function for WUS/WOX5 factors to suppress differentiation is conserved in all euphyllophytes, but that special functions of WOX5 involving cell-to-cell movement between QC and columella have evolved in the seed plant lineage (Zhang et al., 2017). Thus, it is conceivable that following the polyploidization event that took place in the seed plant lineage (Jiao et al., 2011), a duplication of an already established genetic circuit allowed diverging in activity patterns, with one circuit regulating SAM and the other RAM maintenance. However, because *CrWUL* is active both in the fern RAM and SAM (Nardmann and Werr, 2012), the hypothesis, stating that the WOX5 circuit evolved from a SAM regulatory WUS circuit to control RAM, is maybe less likely. Instead, the WUS/WOX5 ability to promote stem cell maintenance under the control by peptide-mediated receptor kinase signaling with a potential for directional signaling could have been an efficient way of positioning a stem cell niche, that therefore would have been co-opted in different contexts during evolution. Indeed, WOX4, a more distant paralog still belonging to the same major clade as WUS/WOX5, similarly maintains the cambial stem cell niche. WOX4 is regulated by a CLE peptide (CLE41/TDIF) emanating from the phloem side of the cambium, sensed by a receptor kinase (PXY/TDR) in the cambium (Etchells et al., 2016). Thus, in common, there is a directional peptide signaling positioning the central region of a stem cell niche. The components for homologous peptide/receptor kinase signaling appear conserved in land plants (Nikonorova et al., 2015). It will be very interesting to know if CrWUL is similarly controlled by peptide/receptor kinase signaling, or if this evolved in the seed plant lineage.

In a recent opinion paper, Liu and Xu (2018) discuss the potential importance of a seed plant specific gene duplication of other paralogous WOX genes, belonging to the “intermediate clade WOX” (IC-WOX; Hedman et al., 2013), for the allorhizoic root evolution in seed plants. IC-WOX genes are found in vascular plants (Liu and Xu, 2018). In the fern *C. richardii*, the IC-WOX homolog expresses specifically and transiently in root founder cells, during lateral and adventitious root development, suggesting that it is critical for root initiation (Nardmann and Werr, 2012). Similarly, its co-orthologs, *AtWOX11/12* expresses specifically in root founder cells during adventitious rooting (Hu and Xu, 2016; Liu et al., 2014). Intriguingly, the paralogs *AtWOX8/9* instead express specifically in the hypophyseal cell of the embryo, which gives rise to the QC and columella precursors of the seed plant primary/allorhizoic root (Breuninger et al., 2008). In both the adventitious roots and the embryonic root meristem *WOX5* expression is initiated at a slightly later stage. Thus, Liu and Xu (2018) proposes that the gene duplication within the IC-WOX clade, which resulted in the *WOX11/12* genes that retained activity in adventitious root initiation and the *WOX8/9* genes that evolved a novel activity specifying an embryonic cell as a root founder cell, may have paved the way for a new type of root meristem giving rise to the allorhizoic/primary root.

## The Root Cap – Protecting, Sensing, and Signaling

The RAM is maintained at the tip of the root, and as the root penetrates the soil there is an apparent risk of damaging this delicate structure. Hence, there most likely has been a strong selection force for the evolution of a structure protecting the RAM. Indeed, in all extant vascular plants, both euphyllophytes and lycophytes, the RAM is protected by a root cap (Kumpf and Nowack, 2015). The root cap consists of a columella and LRC. It facilitates the root’s growth in soil due to production of mucilage, exudes various molecules, and may release long-lived cells into the rhizosphere to repel pathogens and attract symbionts (Kumpf and Nowack, 2015). Moreover, the root cap functions as a gravity-sensing organ rendering positive gravitropism to the root. For this purpose, the columella, in some plants also the LRC, harbors starch-containing amyloplasts, also called statoliths. The statoliths sediment with the gravity vector, which is sensed by the cell. In response, auxin flux in the RAM is modified, resulting in differential elongation of cells, and consequentially bending of the root tip with the gravity vector (Su et al., 2017). Furthermore, the LRC contributes hormonal cues that specifies cells competent for branching, thereby influencing how the entire root system may develop (Xuan et al., 2016). Thus, the root cap is a vital organ for the plant. This is consistent with the early and independent evolution of a root cap in lycophytes as well as in euphyllophytes.

There is quite a large variation in how the root cap is organized within root meristems of different species, and a number of different types have been described. The root cap can be clearly delimited from the QC and has its own stem cells as in Arabidopsis, which is therefore said to have a “closed” configuration. In other plants, such as in pea, the root cap initial cells are not clearly distinguishable from the QC and those meristems are said to be “open” (Kumpf and Nowack, 2015). Gymnosperm meristems are open with no clear boundary between root cap initials and QC. Furthermore, in certain lycophytes, as in ferns, there is a single apical cell dividing in a distinct pattern to give rise to all cell types of the root, including the root cap, while in other lycophytes the meristem structure more resembles that of seed plants, and they can be either open or closed (Fujinami et al., 2017). A remarkably well preserved fossil of a progymnosperm with an active root meristem displays a clearly identifiable root cap surrounding a very broad meristem (Hetherington et al., 2016). Tracing domains of clonally related cells suggested that this carboniferous root meristem differed in organization from any previously described root meristem organization type. Hence, although extant plants display quite a variation in meristem/root cap organization an even greater diversity is likely to have existed in extinct plants. Intriguingly, all must be or must have been able to accommodate for a constant replenishing of their root caps.

As the root grows through the soil, the root cap cells are sloughed off or become released from the LRC *via* programmed cell death (Fendrych et al., 2014; Kumpf and Nowack, 2015). Hence, there is a need to produced new columella and LRC cells at the same rate as cells are lost. In Arabidopsis, the activity of WOX5, required for columella stem cell fate (Sarkar et al., 2007), is balanced by the activity of a TF of the NAC-family, called FEZ, which instead promotes the formation of LRC initials (Willemsen et al., 2008). FEZ also activates another NAC TF, called SOMBRERO (SMB), which together with BEARSKIN1 and 2 promotes the differentiation of LRC cells. SMB in turn represses *FEZ* to prevent overproduction of LRC cells, while WOX5 represses *SMB*, thereby controlling a precise development of the columella and LRC (Bennett et al., 2010, 2014). Despite the evolutionary importance of the root cap, surprisingly few studies exploring putative key genetic circuits shaping root cap development have been carried out in a comparative context. Recently, a transcriptome analysis of *S. moellendorffii* roots, comparing transcripts of the DZ and the EDZ, identified genes related to FEZ to be active in the roots of this lycophyte (**Figure 2C**; Huang and Schiefelbein, 2015). Detailed expression analyses and functional studies of these homologs may help us further understand the evolution of the root cap.

## Cell-to-Cell Signaling Determines the Patterning of the Stele

A vascular system with xylem and phloem that provides efficient transport of water, mineral nutrients, sugars, hormones, and other signaling molecules was a key evolutionary innovation, predating the evolution of true roots (**Figure 1**; Kenrick and Strullu-Derrien, 2014; Taylor et al., 2009). As the root meristem generates cells within the DZ, these cells acquire specific identities, primarily depending on their position relative to each other (Yu et al., 2017). The procambium, the meristematic tissue from which the primary vascular tissues are derived, is localized in the center surrounded by the ground and dermal tissues. Within the procambium xylem and phloem precursor cells are patterned in a species-specific manner, in most roots in a protostele arrangement. In a protostele, xylem forms in the center and may be arching out in distinct patterns, flanked by procambium and phloem. This pattern is established already in the embryo and propagated by the RAM (De Rybel et al., 2016). In Arabidopsis, the stele has a diarch arrangement, i.e., an axis of xylem traverses the stele. Within the stele, two types of primary xylem vessels are formed: protoxylem with spiral or annual secondary cell walls, and metaxylem with reticulated or pitted walls. Protoxylem develops at the periphery of the axis, while metaxylem later differentiates at the center of the stele. This is an exarch pattern. Also in lycophyte roots, a protostele is common, but here the xylem pattern is opposite to most other plants, and protoxylem forms in the center and metaxylem toward the periphery (endarch pattern). In certain lycophyte roots, the endarch vascular tissues instead surrounds a pith, in a siphonostele arrangement. In monocots, multiple xylem strands may surround a pith, but as in other angiosperms, the xylem forms in an exarch pattern, with metaxylem toward the pith and protoxylem toward endodermis (Taylor et al., 2009).

**FIGURE 3:**
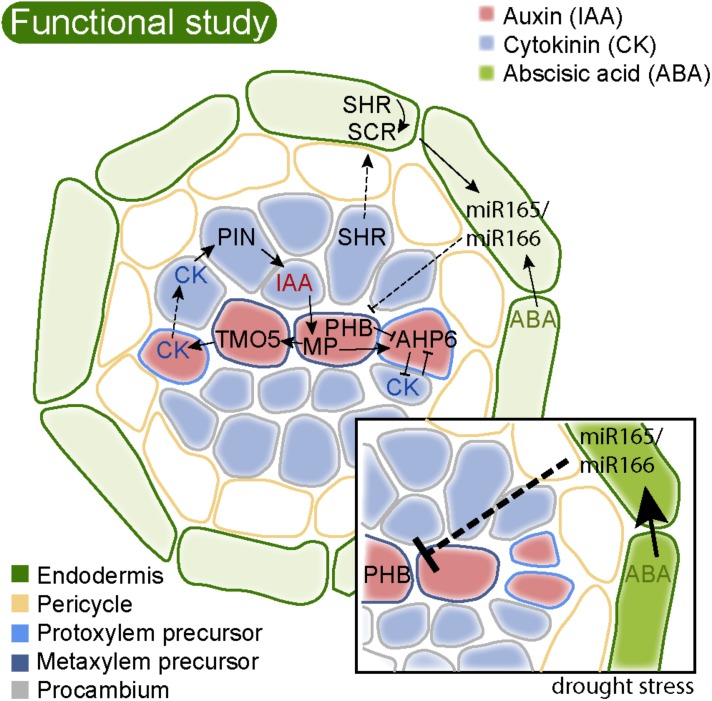
Regulatory circuits of highly conserved hormones and genes regulate Arabidopsis root stele patterning. The cartoon shows a cross section of the stele surrounded by the endodermis just above the vascular initials/stem cells. Different cell identities are indicated by colored cell walls. The xylem axis is specified by a focused auxin (IAA, red) maximum. This is a result of lateral PIN-mediated transport of IAA from procambial cells to the xylem axis (Bishopp et al., 2011). In the xylem axis, IAA activates *MP*, which in turn activates *TMO5*. TMO5 activates *LOG4*, encoding the last step in cytokinin (CK) biosynthesis (De Rybel et al., 2014; Ohashi-Ito et al., 2014). Cytokinin is not sensed in the xylem axis but moves to the procambium. Here, it triggers cell division, as well as activation of PINs for lateral IAA transport to the xylem axis. MP in the axis also activates *AHP6*, and AHP6 negatively interferes with CK sensitivity, required for proper protoxylem cell identity (Bishopp et al., 2011). PHB is transcribed throughout the stele, in an IAA biosynthesis dependent manner (Ursache et al., 2014). SHR is also transcribed in the stele, but the SHR protein moves out to the endodermis (Nakajima et al., 2001). Here it activates SCR, and together they activate a set of genes for miR165 and miR166. These miRNAs then move back into the stele to restrict PHB mRNA from the stele periphery, and thereby focus PHB activity to the central stele. PHB along with other HD-ZIP III TFs dose dependently determine proto- and metaxylem cell identity (Carlsbecker et al., 2010). Activation of miR165 and 166 also requires basic levels of ABA (Ramachandran et al., 2018). Upon drought stress (inset), ABA levels are increased enhancing miR165 levels, resulting in reduced PHB levels, which consequently shifts xylem cell identity toward formation of more protoxylem cells and less metaxylem. Arrows indicate positive and blocked arrows negative interactions. Dashed arrows indicate cell-to-cell movement.

The vascular pattern in Arabidopsis is determined by a number of evolutionarily conserved factors, including hormones and TFs. Auxin synthesis, transport, and signaling are required for the establishment of the central stele (De Rybel et al., 2016). Auxin is needed to break the initial radial symmetry of the root with the formation of a xylem axis (De Rybel et al., 2014; Bishopp et al., 2011). This process is reinforced by the antagonistic action of cytokinin, which in turn is required for cell proliferation in the neighboring procambium. High auxin levels triggers activation of the AUXIN RESPONSE FACTOR 5 (ARF5)/MONOPTEROS (MP). MP in turn activates TARGET OF MONOPTEROS5 (TMO5) in the xylem axis (Schlereth et al., 2010). TMO5 then directly activates cytokinin biosynthesis (De Rybel et al., 2014; Ohashi-Ito et al., 2014). The presumably high cytokinin level is not sensed within the xylem axis, but instead in the neighboring procambial cells, to which it diffuses or is transported. In the procambium, cytokinin promotes cell division, but it also promotes auxin transporters (PINs) that move auxin toward the xylem axis (Bishopp et al., 2011). The high auxin level in the xylem axis also activates a specific cytokinin signaling component, AHP6, which instead of transmitting the cytokinin signal acts to inhibit it (Bishopp et al., 2011). Hence, in Arabidopsis there is a mutually inhibitory action of auxin and cytokinin which is defining the root vascular pattern (**Figure 3**). If any of these components are disturbed, this will alter the pattern of xylem and procambium in the stele; in particular, the protoxylem is sensitive to perturbations of auxin and cytokinin (Bishopp et al., 2011).

Patterning of the root xylem axis with peripheral protoxylem and central metaxylem requires TFs of the class III homeo-domain leucine zipper (HD-ZIP III) family, regulated by miR165 and miR166 (Carlsbecker et al., 2010; Miyashima et al., 2011, reviewed in Ramachandran et al., 2017). The HD-ZIP III genes as well as miR166 have homologs in all land plants (Floyd and Bowman, 2004, 2006; Prigge and Clark, 2006). In moss gametophytes, HD-ZIP III TFs regulate leaf development (Yip et al., 2016). Because bryophytes are non-vascular plants, these factors must have been recruited to regulate vascular development during the evolution of vascular tissues. Indeed, HD-ZIP III expression is detected in vascular tissues in lycophytes (Floyd and Bowman, 2006; Prigge and Clark, 2006). In Arabidopsis, the HD-ZIP III family includes five members, and mutant phenotypes suggest that they dose dependently specify the xylem cell type identity. Plants lacking all five HD-ZIP III transcription factors fail to develop xylem (Carlsbecker et al., 2010). The miR165/166 regulating HD-ZIP III in the root are produced in the endodermis where they are activated by SHR together with its paralog SCR. SHR is produced in the stele but moves out to the endodermis to activate SCR (Helariutta et al., 2000). Together, these TFs induce the expression of genes coding for miR165 and miR166, which in turn move back into the stele. At the peripheral stele, high levels of miR165 and miR166 strongly reduces the abundance of HD-ZIP III mRNA, in particular of PHABULOSA (PHB). The relatively low HD-ZIP III protein level determines protoxylem cell identity, while less miR165/166 in the center allow high levels of HD-ZIP III TFs governing metaxylem formation (**Figure 3**; Carlsbecker et al., 2010; Miyashima et al., 2011). The HD-ZIP III TFs are tightly interlinked with auxin and cytokinin signaling. All HD-ZIP III genes are directly or indirectly requiring auxin biosynthesis for their transcriptional activation (Ursache et al., 2014), and in turn they modulate both auxin and cytokinin signaling and synthesis components (Carlsbecker et al., 2010; Dello Ioio et al., 2012; Müller et al., 2016). Because of the complex interactions between hormones, TFs and small RNAs, mathematical modeling have been employed to assess what components and parameters are required for reaching a protostele pattern with a traversing xylem axis with peripheral protoxylem and central metaxylem (Mellor et al., 2017).

Although the vascular pattern is inherently distinctive for distinct species, it also appears to be plastic within a species to some degree, allowing endogenous and externals cues to modify the pattern. Abiotic stress, such as drought, results in a vascular pattern with extra protoxylem strands flanking the central metaxylem in Arabidopsis (Ramachandran et al., 2018). Drought stress is mediated by the hormone abscisic acid, ABA, and ABA applications result in a similar pattern as drought. This pattern is similar to lower order HD-ZIP III mutants, and indeed, elevated ABA cause increases in miR165, resulting in reduced HD-ZIP III levels (**Figure 3**, inset; Ramachandran et al., 2018). The levels of miR165 and miR166 have been found to vary with external conditions in a variety of species (Zhao et al., 2007; Liu et al., 2008; Ding et al., 2009) suggesting that modulation of their activity has the potential to change developmental patterning also in these species. It will be very interesting to see if these factors and the auxin/cytokinin balance may underlie the distinct vascular patterning of various species. Among the angiosperms the monocots display a rather different arrangement, with a siphonostele. Importantly, there was a recent report of fluorescent auxin and cytokinin signaling reporters in barley plants, allowing live tracking of hormonal signaling (Kirschner et al., 2018). Such an approach will be needed to understand if and how auxin and cytokinin pattern also the monocot root vasculature. It is obvious that polar auxin transport plays an important role in vascular development in extant plants, but fossil evidence also suggest it was important in now extinct plants. Stunningly, analyses of wood of fossil plants of both arborescent lycophytes and progymnosperms reveal a circular pattern of treachery elements above buds and branch junctions in stems (Rothwell et al., 2009). Such patterns are also seen in extant trees and emanates from routes of polar auxin transport. Thus, this provides evidence for polar auxin transport in vascular tissue formation in 375 million years old lycophytes, and suggests that canalization of auxin was coupled to the evolution of vasculature tissues (Rothwell et al., 2009). Recently, Zhu et al. analyzed the transcriptome of *S. moellendorffii* stems and found that many key factors, such as SHR/SCR, HD-ZIP III, and TMO5, have homologs in this lycophyte although certain components of the gene regulatory network required for Arabidopsis root vascular patterning were not identified. Thus, the regulatory mechanisms of lycophyte vascular development is perhaps less complex or involves different components than in flowering plants (Zhu et al., 2017). Modeling may generate hypotheses for how patterns such as the siphonostele, or the endarch protostele pattern of lycophytes are established. Such hypotheses may be tested by mapping gene expression and regulatory networks in lycophytes and other phylogenetically informative species.

## The Stele Ensures it is Surrounded by a Single Guarding Endodermal Layer

The central function for the root is to take up water and mineral nutrients. In this process, the endodermis forms an apoplastic barrier with the Casparian strip and suberin lamellae restricting diffusion of water nutrients, and thereby allowing a controlled uptake (for reviews, see Geldner, 2013; Barberon and Geldner, 2014). This very specialized cell layer likely evolved at least twice, in the lycophytes and in the euphyllophytes. Fossils of early species of each of these two lineages apparently lacked an endodermal layer, and extant *Lycopodium* does not have a root endodermal layer (Raven and Edwards, 2001; Kenrick and Strullu-Derrien, 2014; Raven, 2018). Hence, the endodermis may have evolved as a relatively recent innovation in each lineage (**Figure 1**). The endodermis is the inner layer of the ground tissue of the root, which outside of the endodermis harbors the cortex. The cortex generally consists of parenchymatic cells and can provide several different functions such as storage or, by the formation of aerenchyma, means to improve flooding tolerance. The outer layer of the cortex may also develop an exodermis, a first barrier inside the epidermis (Kim et al., 2018).

**FIGURE 4:**
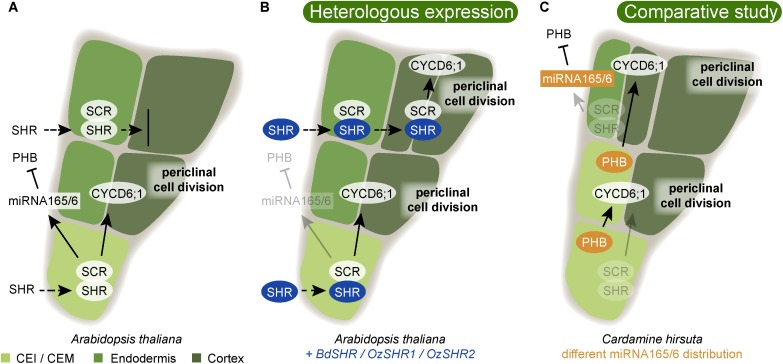
Variability in ground tissue layer number is guided by conserved regulatory mechanisms. **(A)** In Arabidopsis, the cortex/endodermis initial stem cell (CEI) undergoes an asymmetric periclinal cell division to give rise to endodermis and cortex. This is controlled by the movement of the transcription factor SHORTROOT (SHR) into the CEI (Nakajima et al., 2001). Here, SHR becomes nuclear localized and is prevented from further movement to neighboring outer cell layer by complexing with SCR. In the CEI, SHR directly activates transcription of the D-type cyclin CYCD6;1, which is required for its specific cell division (Sozzani et al., 2010). A correct patterning with one endodermis and one cortex cell layer also requires the restriction of PHABULOSA (PHB) to the stele by the action of the endodermally expressed miRNA165/6 (Miyashima et al., 2011). **(B)** When *SHR* homologs from *Brachypodium distachyon* (*BdSHR*) or *Oryza sativa*, rice (*OzSHR1*/ *OzSHR2*) are heterologously expressed in Arabidopsis, the SHR protein moves beyond the neighboring endodermis, and induces an extra cortical cell layer (Wu et al., 2014). Blue highlights indicate heterologously expressed proteins. **(C)** In *Cardamine hirsuta*, PHB is not restricted to the stele due to a different miRNA165/6 activity domain. The expanded PHB activity domain results in the formation of a cortex/endodermis mixed (CEM) cell. Here, PHB activates *CYCD6;1* resulting in a division, and thereby the formation of an additional cortex cell layer (Di Ruocco et al., 2018). Orange highlights indicate differences in expression domains compared to Arabidopsis. Arrows indicate positive interaction, and blocked arrows indicate negative interaction. Dashed arrows indicate cell-to-cell movement.

In all plants that have an endodermis, there is only one layer, just next to the stele. Thus, genetic mechanisms have to operate to delimit the specific differentiation to only this ground tissue layer. The prevailing hypothesis of how plants ensure the formation of a single endodermal layer just outside the stele relies on molecular communication from the stele providing both positional information and information for endodermal differentiation (Wu et al., 2014; Doblas et al., 2017b). In Arabidopsis, both ground tissue layers, cortex and endodermis, originate from the same cortex/endodermis initial stem cell (CEI) which first divides anticlinally. The daughter cell then undergoes an asymmetric periclinal division to produce one endodermal and one cortical cell layer (**Figure 4A**). If either SHR or SCR is mutated, this periclinal division does not occur, and only one ground tissue layer is formed (Di Laurenzio et al., 1996; Helariutta et al., 2000). In the *shr* mutant, this layer exhibits cortex characteristics suggesting that SHR is required for endodermis differentiation (Helariutta et al., 2000), while in the *scr* mutant, the single layer exhibits a mix between cortex and endodermis characteristics (Di Laurenzio et al., 1996). Important for the positioning of the endodermis just outside of the stele is the movement of SHR into the neighboring outer cell layer from the stele, where it is expressed (Nakajima et al., 2001). Here, SHR activates SCR, with which it forms a complex in the nucleus (Cui et al., 2007; Welch et al., 2007). This prevents SHR from moving further and prevents additional periclinal cell divisions thereby ensuring the formation of only one endodermal layer. Together, SHR and SCR trigger the asymmetric periclinal cell division resulting in the endodermal and cortex layers by direct induction of *cyclin D6;1* (*CYCD6;1*) (**Figure 4A**; Sozzani et al., 2010).

Wu et al. (2014) tested the potentially conserved functions of monocot SHR by introducing SHR homologs from *Brachypodium distachyon*, *BdSHR*, and *Oryza sativa* (rice), *OsSHR1*, and *OsSHR2*, into Arabidopsis (**Figure 4B**). As expected, both BdSHR and OsSHR1/2 were able to activate and bind to Arabidopsis SCR. However, the movement of the SHR homologs was not restricted to one layer, but they continued moving, triggering the formation of additional cortex, but not endodermal layers (**Figure 4B**; Wu et al., 2014). Thus, this finding may uncover a potentially important role for SHR/SCR to trigger multiple cortex divisions. It is likely that this is an important mechanism in monocots that often have many cortex layers. This experiment also revealed that SHR alone is not sufficient to induce endodermis differentiation. Instead, additional conserved signals from the stele together with SHR are likely required for determination of a single endodermal layer. SHR and SCR are highly conserved. In conifers, the *Pinus sylvestris (PsySCR*) homolog is specifically expressed in the endodermis and the ground tissue initials (Laajanen et al., 2007) and also SHR homologs have been found in conifer roots (Solé et al., 2008). Going even further back the land plant phylogeny, Zhu et al. (2017) found SHR and SCR homologs in the transcriptome of *S. moellendorffii* roots, stems, and leaves. However, the presence of homologous genes might be a good first indication but does not necessarily mean that the function is conserved as well. For instance, SCR and SHR homologs were also found to be essential for bundle sheath specification in leaves (Cui et al., 2014; Yoon et al., 2016). Thus, more detailed analyses are required to test the hypothesis that these homologs perform similar functions as their Arabidopsis homologs.

The differentiation of the endodermis involves the formation of a Casparian strip, specific depositions of lignin in the cell wall between the endodermal cells, providing an apoplastic barrier. Next step is incorporation of a suberin-containing lamellae in the wall, while specific cells are passage cells and are kept open for intake of molecules (Geldner, 2013; Doblas et al., 2017a; Andersen et al., 2018). In this process, SHR acts at the top of a gene regulatory cascade, and directly activates another key TF, MYB36, and these two TFs activates genes for both Casparian strip and suberin lamellae differentiation (Kamiya et al., 2015; Liberman et al., 2015). Interestingly, a stele derived peptide, which signals into the endodermal layer, ensures proper maintenance of the Casparian strip, providing additional molecular surveillance from the stele on the endodermis (Doblas et al., 2017b). Recently, a study found that the genetic regulation of endodermis formation is highly conserved in tomato (*Solanum lycopersicon*) (Li et al., 2018), but the genetic regulation of endodermis differentiation might be conserved also outside of angiosperms. Indeed, phylogenetic analyses could identify homologs to many other key factors in all plants with an endodermis (Li et al., 2018). However, although CASP proteins, responsible for localization of the Casparian strip, are highly conserved among plants, only euphyllophytes have CASPs with a specific protein domain important for their function (Roppolo et al., 2014). Continued research into evolutionary aspects of the components now rapidly being discovered in Arabidopsis promises to shed light on endodermis evolution within a close future.

While the function of the endodermis as a barrier for water and nutrient uptake is well established, the purpose of varying amounts of cortex layers is less obvious. Upon the observation that cortex proliferation can be induced by oxidative stress, Cui (2015) speculated that cortex proliferation might be a protective mechanism against abiotic stress. On the other hand, there is evidence for a trend in plant evolution to produce thinner roots, presumably to improve the efficiency of soil exploration and to reduce the dependence on symbiotic mycorrhiza (Ma et al., 2018). Accordingly, a reduced root cortical cell file number in maize was correlating with improved drought tolerance (Chimungu et al., 2014). A recent study identified a mechanism for generating multiple cortex layers in *Cardamine hirsuta*, a close relative of Arabidopsis. Di Ruocco et al. (2018) showed that levels of miR165/166 not only are important for vascular development (see above) but also for the determination of cortical cell number in *C. hirsuta*. Enhanced *MIR165A* expression causes the formation of only one instead of two cortical cell layer, while reduced miR165/6 activity in Arabidopsis caused additional formation of cortex layers by an expanded expression domain of PHB triggering CYCD6;1 activation, similar to SHR/SCR (**Figure 4C**; Di Ruocco et al., 2018). Thus, small changes in miRNA activity can have a big impact on root anatomy and may underlie anatomical differences between species.

## Outlook

As we have seen, we have quite a detailed understanding on how morphogenesis and anatomy of the Arabidopsis root is established. How the broadly similar morphology and anatomy of distantly related monocot, gymnosperm, fern, or lycophyte roots are genetically controlled is, however, largely unknown. A better understanding of the underlying genetic regulation will allow us to view the evolution of roots in a clearer light. Although roots are essential for almost all vascular plants, for agriculture, and for ecosystems we have a rather limited understanding of how this essential organ has evolved, but also how its development is regulated in most species. Hence, despite the importance of roots, there are quite a few outstanding questions remaining to be answered. Has the root evolved as a modified shoot, as the presence of homologous regulatory factors may suggest. Or is the root an entirely novel organ, as the opposite auxin transport patterns in the shoot and root meristem indicates. Is the primary, allorhizous, seed plant root homologous with the adventitious, homorhizous, roots of ferns? Is there “deep homology” as potentially indicated by the identification of putative homologs to key root development regulators in lycophytes? How could complex and central structures for root function such as the root cap and the endodermis have evolved independently both in lycophyte and euphyllophyte roots? How did the intricate cell-to-cell communication required for root patterning evolve?

Addressing these and other questions will be facilitated by the very rapid technology development and data generation from next-generation sequencing approaches. Current efforts in characterizing transcriptome and genome sequences of the lycophyte *S. moellendorffii* (Banks et al., 2011), several fern species, including *C. richardii* (Wolf et al., 2015), the conifer *Picea abies* (Nystedt et al., 2013) in addition to the vast amount of data that is accumulating for non-vascular “outgroup” plants, such as the moss *Physcomitrella patens* (Rensing et al., 2008) and the liverwort *Marchantia polymorpha* (Bowman et al., 2017), are providing information on genetic advances that occurred during land plant’s first evolutionary steps as well as when the seed plants evolved, and beyond. Initiatives to sequence the genomes of yet a large number of phylogenetically important vascular plants, both non-seed plant and seed plants, within the 10K initiative, which is leveraging the 1K effort of sequencing 1000 plant genomes (Cheng et al., 2018), will most likely substantially contribute to illuminating various aspects of how roots may have evolved.

At the same time as we are exploring the vast diversity among species and their morphologies and anatomies, it will be important to develop non-angiosperm models of vascular plants (Schulz et al., 2010). Models allow building of knowledge within a research community, for detailed comparative studies with non-model plants by various approaches. It will be essential to establish protocols for transformation of plants to allow reverse genetics. Currently, there is an efficient transformation protocol for the fern *C. richardii* (Plackett et al., 2014), *A. filiculoides* is emerging as another rapidly growing fern model with great potential (de Vries and de Vries, 2018), several species of *Selaginella* are emerging lycopod models (Schulz et al., 2010), and transformation protocols and various resources exist for the conifer *P. abies* (Uddenberg et al., 2015). Furthermore, in a model species detailed gene expression analyses using laser capture microdissection coupled to RNA sequencing, or even single cell approaches, are feasible, and will provide opportunities to build detailed gene expression maps. This will be instrumental for co-expression analyses and construction of gene regulatory networks. Such networks can be compared with the detailed gene regulatory network around key developmental regulators in Arabidopsis (Taylor-Teeples et al., 2014; Santuari et al., 2016; Drapek et al., 2017) to allow inferences of important shifts potentially underlying evolutionary novelties. Together with hormone signaling localization and detailed morphological and anatomical studies of potential changes resulting from external signaling or perturbation, it would allow inferring developmental core modules responsible for specific features. In such a system meaningful heterologous complementation experiments can be conducted with key genes from closely or distantly related species, to test conservation of protein function. In **Figures 2**–**4** we point out various approaches by which knowledge of a process in the model plant Arabidopsis can be used to widen our understanding of similar processes in other plants. With established fern and lycopod models we can extend this type of analyses substantially. Along with transcriptome data from a dense phylogenetic sampling, we are on the way to a comprehensive understanding of the underlying genetic key factors for morphological features such as the RAM, root cap, endodermis, or specific stele patterns. Mirroring morphological and anatomical outcomes of genetic and hormonal perturbation experiments with the phenotypes of extant, but also extinct fossil morphologies and anatomies, will allow us to formulate specific and testable hypotheses on how genetic networks may be rewired during evolution to generate novel morphologies, or even novel organs – such as the repeated evolution of roots. There are indeed exciting times ahead when we dig deeper into the evolution and developmental biology of plant roots.

## Author Contributions

FA and AC prepared and finalized the manuscript together.

## Conflict of Interest Statement

The authors declare that the research was conducted in the absence of any commercial or financial relationships that could be construed as a potential conflict of interest.
